# A National Survey Evaluating the Impact of the COVID-19 Pandemic on Students Pursuing Careers in Neurosurgery

**DOI:** 10.3390/neurosci2040023

**Published:** 2021-10-03

**Authors:** Roxanna M. Garcia, Rebecca A. Reynolds, Hannah K. Weiss, Nathan A. Shlobin, Lola B. Chambless, Sandi Lam, Nader S. Dahdaleh, Gail Rosseau

**Affiliations:** 1 Department of Neurological Surgery, School of Medicine, Northwestern University Feinberg, Chicago, IL 60611, USA;; 2 Department of Neurological Surgery, Vanderbilt University Medical Center, Nashville, TN 37235, USA;; 3 Division of Pediatric Neurosurgery, Ann and Robert H. Lurie Children’s Hospital, Chicago, IL 60611, USA; 4 Department of Neurosurgery, School of Medicine and Health Sciences, George Washington University, Washington, DC 20037, USA

**Keywords:** COVID-19, pandemics, education, medical, graduate, students, medical, neurosurgery, internship and residency

## Abstract

**Background:**

The COVID-19 pandemic has profoundly disrupted medical education and the residency application process.

**Methods:**

We conducted a descriptive observational study in April 2020 of medical students and foreign medical graduates considering or pursuing careers in neurosurgery in the United States to examine the impact of the pandemic.

**Results:**

A total of 379 respondents from 67 medical schools completed the survey. Across all participants, 92% (n = 347) stopped in-person didactic education, and 43% (n = 161) experienced basic science and 44% (n = 167) clinical research delays. Sixty percent (n = 227) cited a negative impact on academic productivity. Among first year students, 18% (n = 17) were less likely to pursue a career in neurosurgery. Over half of second year and third year students were likely to delay taking the United States Medical Licensing Examination Steps I and II. Among third year students, 77% (n = 91) reported indefinite postponement of sub-internships, and 43% (n = 53) were unsatisfied with communication from external programs. Many fourth-year students (50%, n = 17) were graduating early to participate in COVID-19-related patient care. Top student-requested support activities included access to student-focused educational webinars and sessions at upcoming conferences.

**Conclusions:**

Medical students pursuing careers in neurosurgery faced unique academic, career, and personal challenges secondary to the pandemic. These challenges may become opportunities for new initiatives guided by professional organizations and residency programs.

## 1. Introduction

These are “unprecedented times” for all, including those involved in medical student education [1]. Although attention during the SARS-CoV2 pandemic has been appropriately focused on providing patient care and educating medical professionals as understanding of the disease and associated treatment recommendations have evolved, substantial efforts have also been directed toward managing urgent and emergent surgical conditions and minimizing collateral damage to surgical education for faculty and residents [2–4]. While medical students (MSs), recent graduates, and upcoming trainees have been identified as vulnerable groups [5], the literature demonstrating the impact of the pandemic on these groups continues to be grow [6–8]. Since the start of the pandemic, its impact on career choices is thought to affect up to one-fifth of medical students [9]. Understanding how to support individuals at early stages of training during the current pandemic is essential in order to continue to recruit, retain, and deploy talented trainees in surgery.

On March 17 2020, the Association of American Medical Colleges (AAMC) issued guidelines recommending a pause in clinical rotations for MSs [8], restricting medical school education to online lectures and virtual didactic sessions to comply with social distancing requirements. Early clinical exposure and direct contact is invaluable for stimulating and strengthening students experience in choosing a specialty and creating the foundation for professional identity, particularly early during training [1,9]. However, the disruption to medical education caused by the pandemic has subsequently limited exposure to clinical medicine due to cessation of clinical rotations and disbanding of in-person group learning, limiting initial exposure to surgical specialties [8,9]. Surgical sub-specialties such as urology, plastic surgery and otolaryngology have documented the multifactorial impact the pandemic has had on residency selection, education, and recruitment. MSs in their first and second years (MS1, MS2, respectively) had delayed or missed their first exposure to surgical specialties, with potential long-term implications for career choice. MS3s and MS4s who have decided to pursue careers in surgery have had questions about navigating the residency match process, including visiting sub-internships, obtaining letters of recommendations, and interviewing for residency positions, in addition to optimal strategies for starting internship.

The primary objective of this study is to expand upon previously reported preliminary findings, to better characterize the impact of the COVID-19 pandemic among students pursuing careers in neurosurgery, including foreign medical students (FMGs), and the beginning of internships of recently matched MSs [10]. Recent work has also shown that this population has significant concerns about learning basic neurosurgical knowledge that will help inform career decisions [11]. The study was created to identify special concerns and educational needs of these groups to inform surgical leadership, catalyze the development of strategies to address the concerns and needs of students directly, and strengthen logistical and psychosocial support networks for the youngest members of surgical fields.

## 2. Materials and Methods

### 2.1. Study Population

We sought to sample all U.S. medical students and U.S.-based FMGs who are considering applying to U.S. neurosurgical programs, as well as those that matched into neurosurgical programs in March 2020. A listing of 2020 U.S. allopathic medical schools was obtained from the AAMC website, members.aamc.org, which includes 161 schools nationally [11]. A search was performed on April 5 2020 to identify publicly available student names associated with neurosurgical student organizations in all U.S. allopathic medical schools (Appendix A). Organizational and individual emails, as well as social media accounts and websites for neurosurgical student organizations, were aggregated. To cross reference these findings, the publicly available version of the American Association of Neurological Surgeons (AANS) Medical Student Chapter Directory and the 2020 Neurosurgical Residency Match list at aans.org was consulted on April 5 2020 [12]. The AANS database contains 150 schools with 2 to 8 participants each. Combining these data with the National Resident Match Program (NRMP) match results (which estimates approximately 350 applications are submitted annually), the target population of medical students is approximately 1400 students. Institutional Review Board approval through Vanderbilt University was obtained, and the survey was deemed exempt status given responses were anonymous and did not contain any protected health information. Respondents consented to participate in the survey after being informed of its content and associated risks and benefits.

### 2.2. Survey Design and Administration

This study is a descriptive observational, cross-sectional study of students aspiring to be neurosurgical trainees in the United States, structured as a purposeful sample. The survey design adhered closely to the Checklist for Reporting Results of Internet E-Surveys (CHERRIES) checklist [13]. Demographic details collected included age, sex, year in school, degree program, presence of home program, presence of home neurosurgery rotation, and presence of a home AANS interest group. Region of medical school was determined by utilizing designations of the United States Census Bureau [14]. Survey questions were created from a review of similar studies and from factors likely to be impacted by the COVID-19 pandemic and relevant to a medical student pursuing a career in neurosurgery [15–17].

A pilot survey was completed by 23 students interested in neurosurgery from four institutions (Northwestern University, Vanderbilt University, George Washington University, and Tufts University). The survey underwent several revisions using feedback on usability and technical functionality. No further validation was performed due to the time constraints early in the pandemic. The final survey was written in Google Forms format (google.com/forms) and consisted of 20 questions distributed to all survey respondents, with an additional eight questions for students who indicated that they had recently matched into a neurosurgical residency program during the 2019–2020 application cycle. The survey description and details were included in the initial survey text, including its length, anonymity, optionality, investigators, and purpose of the study. No incentives were offered. The responses were anonymous to maintain confidentiality. After submission, a separate survey offered students the option to supply the name of their medical school and email address for future communication, but responses could not be linked to the primary survey.

From April 13 to May 8 2020, 236 students identified from public sources were emailed (five times) to voluntarily complete this online survey. In addition, the open survey was distributed on social media (Twitter and Facebook) and Neurosurgery Hub (neurosurgery-hub.org), an open website aimed at trainees interested in or pursuing neurosurgery. The survey was also distributed via the Neurosurgery Program Coordinator ListServ, providing all neurosurgical program coordinators the opportunity to distribute the survey email to medical students at their institution known to be interested in neurosurgery.

### 2.3. Statistical Analysis

Frequency tables are provided to summarize characteristics of the populations and survey responses. A descriptive statistical analysis compared survey responses by MS year using two-sided Student’s t-tests and chi-squared tests for continuous and categorical variables, respectively. When appropriate, Fisher’s exact test was implemented to adjust for small cell counts. Variables with greater than 5% missing values were excluded from the final analysis. *p*-values < 0.05 were considered statistically significant. Stata Inter-cooled, version 14 (Stata Corporation, College Station, TX, USA) was used for statistical analysis. Word clouds were generated using NVIVO Pro, version 12 (QSR International, Melbourne, Australia).

## 3. Results

### 3.1. Study Demographics

A total of 379 respondents completed the online survey. Third-year medical students represented the largest frequency of survey respondents (32%, n = 121). The overall survey response rate was estimated to be 25% averaged across 4 years, with the most samples from MS3s. Thirty-six percent (n = 138) were from the South, and 37% identified as female sex (n = 140). These responses represented at least 67 distinct allopathic medical schools throughout the country. Over 93% (n = 353) reported a Stay-at-Home order from their home medical school. Seventeen percent (n = 62) reported lack of home neurosurgery program. The majority of medical students indicated home neurosurgery rotations were elective (85%, n = 317), with 59% offered in multiple years throughout medical school. A complete summary of the respondent characteristics is shown in Table 1. Among the 54 MS4s, 82% (n = 44) matched this year.

### 3.2. Impact of COVID-19 on Academic Productivity, Research Activities, and Career in Neurosurgery

**Figure 1.**
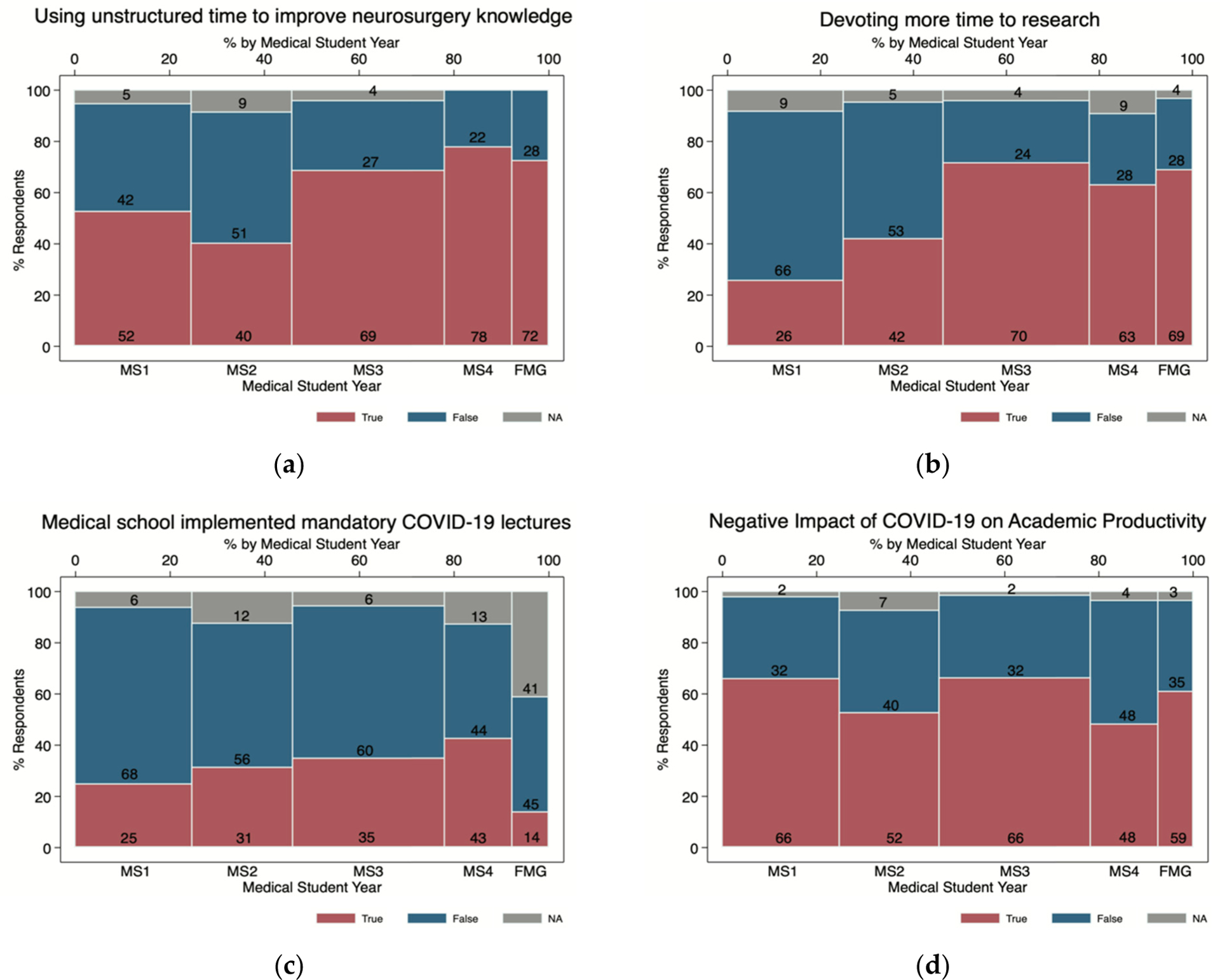
Survey responses (n = 379) among medical students and foreign medical graduates on the overall impact of COVID-19 pandemic. Compressed stacked two-way bar charts showing survey responses by medical student year and foreign medical graduates currently pursuing careers in neurosurgery during the COVID-19 pandemic. (**a**) Using unstructured time to improve neurosurgery knowledge; (**b**) devoting more time to research; (**c**) medical school implemented mandatory COVID-19 lectures; (**d**) negative impact of COVID-19 on productivity.

Across all respondents, 61% (n = 230) reported that their medical schools have indefinitely postponed clinical clerkships and 92% (n = 347) have stopped in-person didactic education. Basic and clinical science research has been delayed or stopped among 43% (n = 161) and 44% (n = 167) of all participants, respectively. Forty-eight percent (n = 182) of respondents cited an inability to present accepted academic work at conferences secondary to the pandemic. Yet, 60% (n = 227) reported using the unstructured time to improve neurosurgical knowledge, with the greatest frequency among MS4s (78%; n = 42; *p* < 0.01; Figure 1a). Students also described devoting more unstructured time to research, with the highest frequency among MS3s (70%, n = 85; *p* < 0.001; Figure 1b), and to volunteerism, with no difference across years (42%, n = 159; *p* = 0.50). With regard to implementation of COVID-19 related didactics, 31% (n = 117) of respondents reported participation in mandatory lectures through local medical schools, lowest among MS1s (25%, n = 23; *p* < 0.001; Figure 1c). Overall, approximately 60% of participants (n = 227) indicated that the pandemic has had a negative impact on academic productivity, with MS1s (n = 62) and MS3s (n = 80) citing the greatest impact (66%; *p* < 0.01; Figure 1d).

**Figure 2.**
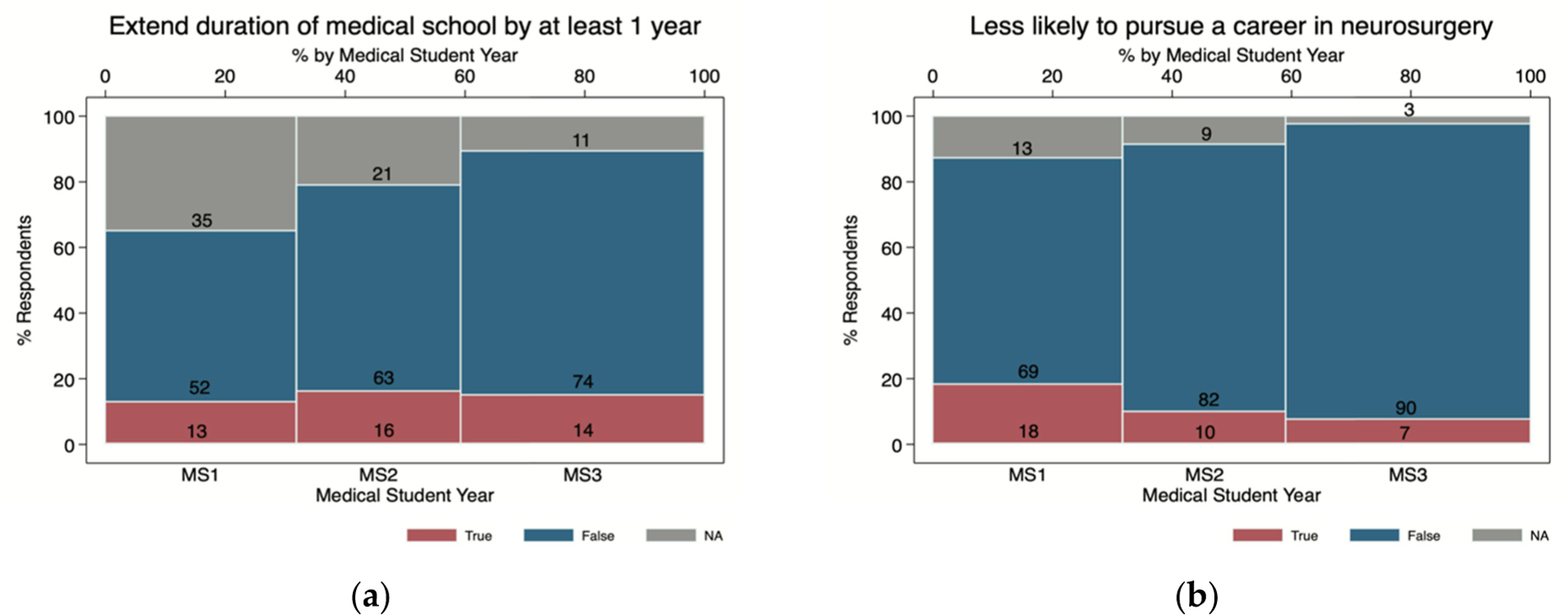
Survey responses from medical students (years 1–3) evaluating the impact of the COVID-19 pandemic. Compressed stacked two-way bar charts showing survey responses on factors that have impacted medical students between one and three years of study (MS1 to MS3) during the COVID-19 pandemic. (**a**) Extend duration of medical school by one year; (**b**) less likely to pursue a career in neurosurgery.

Among MS1s, MS2s, and MS3s, approximately 15% responded they will likely extend the duration of medical school by at least one year, with the biggest effect among MS2s (16%, n = 13; *p* = 0.02; Figure 2a). Furthermore, 18% (n = 17) of MS1s reported that they are less likely to pursue a career in neurosurgery due to the impact of the pandemic and 7% (n = 9) among MS3s (*p* = 0.02; Figure 2b). Fifty-seven percent (n = 46) of MS2s described postponement in taking Step I USMLE and 60% (n = 72) of MS3s indicated they would delay Step II USMLE.

### 3.3. Third- and Fourth-Year Specific Factors

Among the MS3s, approximately 76% (n = 91) reported indefinite postponement of away rotations (sub-internships). Only 36% (n = 43) were satisfied with communication regarding home clinical rotations. Forty-three percent strongly disagreed or disagreed (n = 53) with having satisfactory communication from external programs regarding visiting sub-internships and 31% (n = 37) were dissatisfied with career planning. When asked about their level of concern for the potential of a virtual residency interview in light of travel and social distancing restrictions, 52% (n = 63) responded they would be very concerned or concerned, with only 21% (n = 25) reporting slight or no concern.

Approximately 79% (n = 27) of MS4s who matched into neurosurgery this year reported feeling more concerned about starting an internship during a pandemic. Fifty percent (n = 17) cited graduating from medical school early to participate in clinical care for COVID-19 patients. The mean number of clinical rotations among matched MS4s since July 2019 was 5.2 (SE +/ − 0.3; range: 0–9) with 40% (n = 18) reported satisfactory completion of elective rotations planned for intern year. Conversely, 38% (n = 17) believed that the quality of their medical curriculum was negatively impacted by the pandemic, and many did not believe that they will be able to transition smoothly to a new city (51%, n = 23). Approximately 69% (n = 31) confirmed ability to maintain a satisfactory mental health and well-being, and 60% (n = 27) strongly agreed or agreed that they felt prepared to start residency. The top priorities for MS4s in terms of support from either residency or professional neurosurgical organizations were initiating conversations on how to optimize time spent prior to beginning residency with matched programs (n = 35), receiving online learning opportunities from professional neurosurgical organizations (n = 31), and assistance with finding housing if moving to new city (n = 19).

### 3.4. Foreign Medical Graduate Specific Issues

Twenty-nine FMGs completed the survey. The geographic distribution of FMG respondents represented at least 7 distinct countries or regions (Brazil, Caribbean nation, India, Lebanon, Pakistan, Peru, Ireland, and Saudi Arabia). Among FMGs, over 61% (n = 13) and 78% (n = 18) report delaying Step I and Step II USMLE. None of the respondents matched into neurosurgery this year. Approximately 47% (n = 8) reported they will likely extend the duration of their education at their foreign medical school by at least one year, and 15% (n = 4) reported they are less likely to pursue a career in neurosurgery.

### 3.5. Communication, Immediate Concerns, Perceptions, and Supporting the Future of Neurosurgery

**Figure 3.**
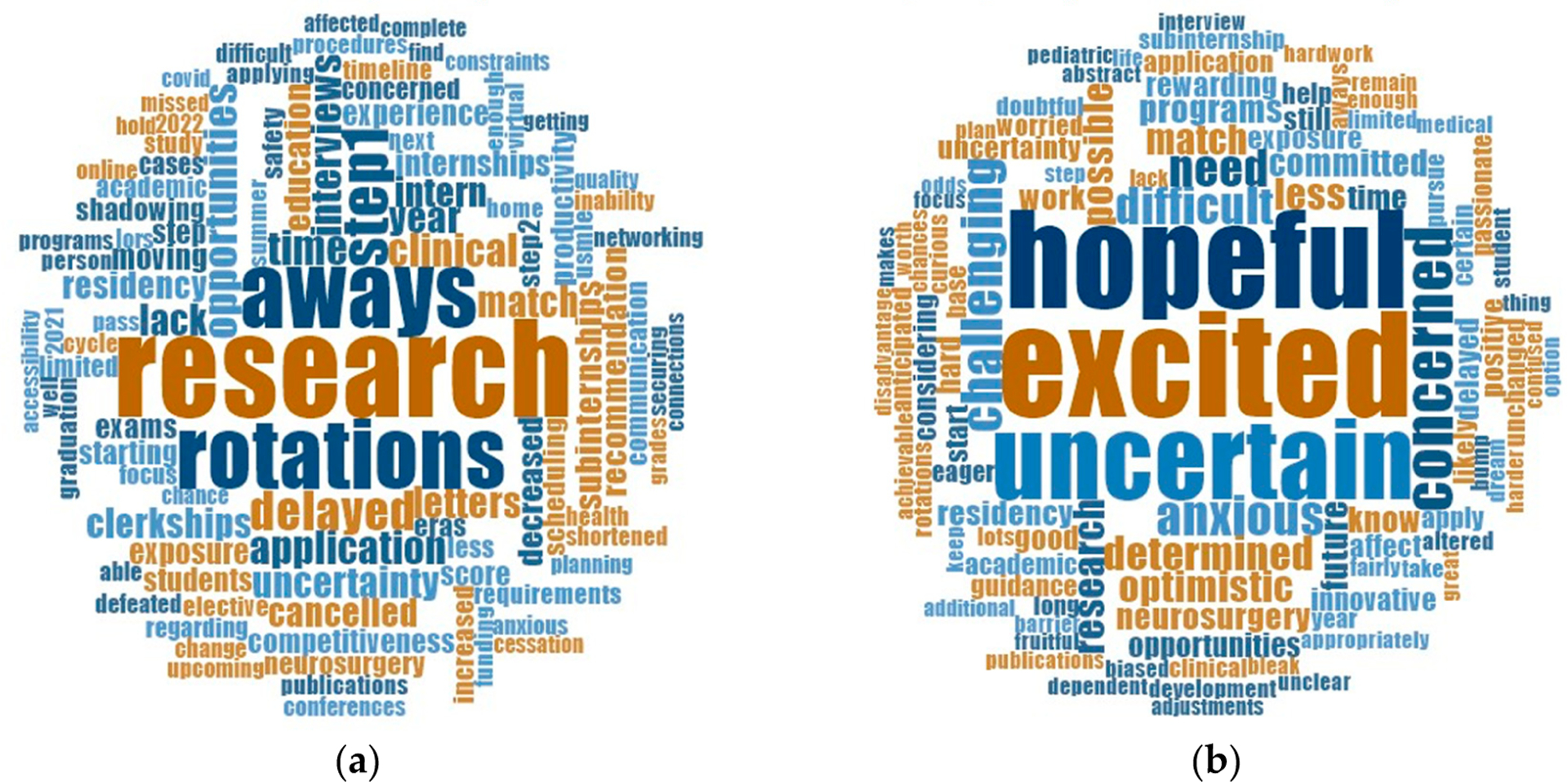
Word clouds generated from medical student and foreign medical graduate respondents pursuing careers in neurosurgery during the COVID-19 pandemic. Word clouds were generated from n = 379 medical students and foreign medical ‘students’ responses to two open-ended questions. The two survey questions were as follows: (**a**) list three words or short phrases [5 word limit] that best describe your ‘immediate concerns’ (**b**) list three words or short phrases [5 word limit] that best describe your ‘thoughts’ about your future in neurosurgery.

Overall, 22% (n = 81) of respondents confirmed satisfaction with communication received related to clinical rotations from home medical programs, with MS3s reporting the highest frequency of dissatisfaction (30%, n = 36; *p* < 0.001). Thirty-one percent of MS3s reported being dissatisfied with communication regarding neurosurgery career planning from home medical programs, followed by MS1s (29%, n = 27; *p* < 0.001). Almost half of all students (45%, n = 167) reported satisfaction with communication and information received from national neurosurgical organizations. Information and guidelines provided by medical programs specific to COVID-19 appeared to be satisfactory to most respondents (60%, n = 165), except for FMGs (48%, n = 9; *p* < 0.001). In response to the complex nature of the pandemic, two open-ended questions were developed to allow participants to report ‘immediate concerns’ related to the pandemic and ‘thoughts’ on their future in neurosurgery. Figure 3a,b capture word clouds generated from these open-ended questions. The top five most common ‘immediate concerns’ in order of frequency were: ‘research’, ‘aways’, ‘rotations’, ‘step1’ [examinations], and ‘delayed’. The top five reported ‘thoughts on their future’ were: ‘excited’, ‘hopeful’, ‘uncertain’, ‘concerned’, and ‘challenging’. Finally, Table 2 summarizes the overall frequency and rank of priorities that could better support students pursuing careers in neurosurgery during the pandemic.

## 4. Discussion

The ramifications of the COVID-19 pandemic among 379 MSs interested in careers in neurosurgery in the U.S. were evaluated through this nationwide survey. This study captured an estimated 25% of medical students interested in career in neurosurgery, and the demographics of all respondents are comparable to the standard cohort applying to neurosurgical residency [18–21]. The results of this survey represent the largest cohort in neurosurgery and findings are relevant to hopeful medical students aspiring to become neurosurgical trainees. While many aspects of clinical work and basic science research related activities have been hindered, students have devised productive ways of using unstructured time to improve neurosurgical knowledge or devote time to augment other research activities. Though concerned (Figure 3b), MSs respondents fortunately remained optimistic and excited about a potential career in neurosurgery (Figure 3b), and many continued to seek guidance and leadership in navigating career planning and educational opportunities within the rapidly changing landscape of the COVID-19 pandemic. We discuss the implications of the survey results by medical school year and highlight possible strategies to help support and encourage students interested in neurosurgery and other surgical specialties during this ongoing, unprecedented disruption in medical education.

### 4.1. Issues and Solutions by Medical School Class

On March 17 2020, the AAMC issued a statement endorsing the closure of all in-person didactic curricula for medical students nationwide [8], albeit without a uniform method of implementing classroom closures. One of the largest surveys available indicated that 1668 U.S. medical students experienced significant repercussions in the residency selection process during the pandemic, secondary to the inability to explore specialties of interest (72.4%), and inability to bolster their residency application (48.1%). Specifically, MS1s and MS2s likely missed valuable educational opportunities, early career exposure important in facilitating interest in surgical subspecialties [9,22,23], and experienced unnecessary stress related to testing delays. Potential alternative experiences for early student exposure may include student-centered webinars or seminar series, informational panels from neurosurgeons and neurosurgical trainees, formal or informal research opportunities, national or regional conferences, or invitations to online departmental weekly conferences and Grand Rounds. Rescheduling USMLE Step examinations was disruptive to learning and scoring well. MS3s struggled to determine how to prioritize their efforts in anticipation of their approaching NRMP application with the indefinite cancellation of clinical rotations and visiting sub-internships, suspension of many research activities, and closing of Pro-metric Testing Centers. The pandemic has exacerbated known anxieties and feelings of unpreparedness in applying to residency including accessing sub-internships, receiving recommendation letters, and determining “perceived goodness of fit” [24–28]. Provision of clear information regarding the Match from the NRMP and neurosurgery professional organizations and creation of opportunities to learn about and interact with programs from departments themselves will assist students in planning for the Match and alleviate anxiety in the process. MS4s conveyed the need for increased communication with programs to allow them to engage in educational and research opportunities and experienced increased concern regarding starting residency and associated personal challenges such as securing housing and relocating family members. Some graduated early to participate in clinical care related to COVID-19. Although the transition has already occurred for M4s surveyed in this study, the authors encourage programs to acknowledge their personal and professional needs and identify clear methodologies to simplify the transition for future MS4s. Notably, these initiatives may continue after the pandemic.

### 4.2. Best Practices and Action Items for Program Directors

During these unprecedented times, Program Directors experienced demands from various levels of institutional, organizational, and governmental leadership, without clear consensus or guidelines. This survey seeks to provide students across all levels of training with a voice to air concerns in order to tailor upcoming programmatic educational and outreach opportunities during the pandemic. At this moment, we must provide unwavering attention to patients suffering from the SARS-CoV2 virus. Meanwhile, we must also recognize the many ramifications of the pandemic on recruitment and retention of bright young physicians. In light of restructured medical education, surgical sub-specialties have devised new, creative ways to engage medical students across all years of training to maximize their potential as a specialty, including virtual rotations and more opportunities to engage with departments. Program directors from otolaryngology stated their evaluation of candidates will likely change, with a shift toward an increased reliance on letters of recommendation, research involvement, and clerkship grades. Transitioning MS4s desired more communication, assistance, and learning opportunities, which have become feasible with the rise of virtual programming and spaces to foster student interest. A coordinated response among surgical subspecialty residency programs, medical schools, and organized surgical specialties is necessary to optimally retain students. These recommendations extend beyond neurosurgery to other surgical specialties in creating a multifaceted action plan to bolster student retention.

### 4.3. Beyond the COVID-19 Pandemic

The COVID-19 pandemic will be transformative. As stated by Rose et al., “this may be a seminal moment in many disciplines in medicine” [1]. While the future is unpredictable, there are many lessons that can be learned from the COVID-19 pandemic and world health crisis and broadly applied to surgical subspecialties. This survey showed that most MS3 applicants are concerned by the prospect of virtual interviews that will appear to continue for at least another year. However, they may be a useful means of candidate assessment, as major successful corporations have found [29], and promote significant cost-savings given alarming expenditures on travel related to residency interviews in recent years [30]. If sub-internships at other institutions continue to be limited, faculty and resident leadership could consider how this may help promote equity during the cost-intensive application process. General surgeons, plastic surgeons, orthopedic surgeons, and otolaryngologists have also proposed this change due to disadvantages for applicants unable to participate in multiple away rotations due to financial means, lack of access, pre-existing health conditions, or inability to travel [31–33]. Plastic surgeons and general surgeons have proposed other strategies to improve the residency match long-term, such as modified core clerkship schedules to better tailor to student interests and to offer virtual opportunities to speak with residents and program directors [31,34]. In the face of the pending Step I USMLE transition to pass/fail rather scoring, residency programs may utilize the COVID-19 pandemic as the stimulus to create a standardized Match process as has previously been endorsed nationally [35,36]. Lastly, widespread efforts to improve medical education and augment standardized residency education and training in low-and-middle-income countries as part of the global neurosurgery movement [37–39] may increasingly gain traction with virtual platforms. More access to online resources and frequent virtual programming through conferences and rotations may supplement in-person learning, appeal to a variety of learner preferences, and democratize medical education [40–46]. Our study provides a closer examination of the concerns of medical students’ interest in or applying into a highly competitive surgical specialty and yields important insights that may be applied to improve medical student education and streamline the residency application process in surgical specialties for years to come. Conversations have often shifted to ‘life with COVID-19′ as opposed to ‘life after COVID-19′; as such, we in surgical education need to consider long term, sustainable approaches to the issues raised in this study.

### 4.4. Study Limitations

While this study should be considered generalizable to medical students interested in neurosurgery in the U.S., it does not apply to an international context. The small number of FMGs reflects the limited generalizability for those completing medical training outside of the U.S. Additionally, these data may not have reached students at institutions without neurosurgical residency programs or interest groups given our survey distribution methodology, with only 17% of respondents reporting no home neurosurgery program. We estimated that our survey represents a response rate of 25% of the target population but this number is difficult to ascertain based on the fluid nature of residency interest over the course of medical training. Additional there was significant differences in total numbers by year. This survey also likely did not capture the MSs that decided to not pursue neurosurgery due to the pandemic and therefore may underestimate some of results. MS with expressed interest in neurosurgery were studied only, so this study may not be universally applicable to all surgical subspecialties. However, many students interested in other surgical subspecialties are facing equivalent challenges with virtual didactic sessions, virtual interviews, and travel restrictions, endowing our findings with applicability to other surgical subspecialties. Our study serves as a baseline assessment that is intended to strengthen support mechanisms for the youngest members of our field and provide guidance to leaders and programs of other surgical specialties in the midst of an undeniably historical moment in public health history.

## 5. Conclusions

The future of surgical fields, such as neurosurgery, depends on continuous recruitment of strong medical students. This survey highlights the gravity of the situation facing these students, particularly students interested in neurosurgery, as medical students at each class level have experienced academic delays, cancellations, and departures from the norm, while its granularity elucidates the specific concerns and needs of medical students. Individuals should endeavor to mitigate disruptions to their education by exploring emerging distance learning. Medical schools, surgical training programs, and organized surgical specialties should consider distinct action items to promote a unified response in addressing these new challenges within their respective fields and devise strategies to augment surgical education in the long-term. Both in spite and due to the COVID-19 pandemic, surgical fields have the opportunity to capitalize upon rapid changes in medical education and the human need for connection to improve outreach and create educational opportunities to develop the next generation of surgeons as those invested in leadership, research, and above all, responsibility to patients.

## Figures and Tables

**Table 1.** Demographic characteristics of medical student survey respondents.

Variable	n = 379	%
**What region of the United States is your medical school located in? (n, %)**		
Midwest	91	24.0
Northeast	84	22.2
South	138	36.4
West	37	9.8
Prefer not to say	6	1.6
Outside United States	23	6.1
	**Sex (n, %)**	
Female	140	36.9
Male	238	62.8
Prefer not to say	1	0.3
Age (mean +/standard error, range)	25.6 (0.1)	19–37
**What year are you currently completing? (n, %)**		
First year (MS1 ^a^)	94	24.8
Second year (MS2)	81	21.4
Third year (MS3)	121	31.9
Fourth year (MS4)	54	14.2
Foreign Medical Graduate	29	7.7
**Are you in a combined, dual degree program? (n, %)**		
No	315	83.1
Yes	63	16.6
**Additional graduate degree type. (n, %)**		
Not applicable	290	81.9
PhD	36	10.2
MA/MS/MPH	24	6.8
MBA	3	0.8
JD	1	0.3
**I am currently under a ‘Stay at Home’ order by my medical school (n, %)**		
False	24	6.4
True	353	93.6
**Do you have a neurosurgery program at your home institution? (n, %)**		
No	62	16.5
Yes	314	83.5
**Does your institution have a neurosurgery rotation for medical students? (n, %)**		
No, we do not have a neurosurgery rotation for students	49	13
Yes, it is optional for students	314	83.3
Yes, it is required for all students to graduate	14	3.7
**Regarding the neurosurgery rotations at your home institution, are they elective, mandatory, or not available?**		
Elective	317	85.0
Mandatory	14	3.8
Not applicable	42	11.3
**Neurosurgery rotation offered multiple years (n, %)**		
Single year	104	27.4
Multiple	224	59.1
Not applicable	51	13.5
**Frequency of neurosurgery rotations by medical school year (n)**		
Year 1	3	-
Year 2	28	-
Year 3	252	-
Year 4	290	-
**Does your university have an AANS ^b^ Medical Student Chapter or other neurosurgery interest group for medical students? (n, %)**		
Yes, AANS	46	12.1
Yes, interest group	264	69.7
No	12	3.2
Do not know	56	14.8

Demographic summary survey respondents (n = 379) collected between April 13th to May 10th of 2020 assessing the impact of the COVID-19 pandemic on medical students and foreign medical students pursuing neurosurgery careers. Abbreviations: ^a^ = MS, medical student; ^b^ = AANS, American Association of Neurological Surgeons.

**Table 2.** Summary of top priorities ranked by frequency by medical student year for how the field of neurosurgery can provide more support during the COVID-19 pandemic.

Priority	MS1 ^a^ n = 94	MS2 n = 81	MS3 n = 121	MS4 n = 54	FMG ^b^ n = 29	Total n = 379	Overall Rank
Student-focused educational webinars	62 (2)	19 (2)	78 (3)	33 (1)	22 (1)	217	1
Focused medical student sessions at upcoming neurosurgical conferences	63 (1)	49 (1)		28 (2)	22 (1)	226	2
Additional away sub-internship spots at programs in 2021	40 (3)	49 (1)			14 (2)	177	3
Additional away sub-internship spots at programs later in 2020			90 (1)	19 (3)		176	4
Delay the match process to allow for away sub-internships			80 (2)			164	5
Be part of a “Future Neurosurgeons” listserv						3	6

Frequencies by column demonstrating the top priorities ranked by medical student year for how the field neurosurgery can provide more support during the COVID-19 pandemic. The rank of the top three by each medical student year is shown in parenthesis (1, 2, or 3). The total frequency and overall rank across all n = 379 participants are summarized in the last column. Of note, open ended fields coded “cancelling away rotations” as the most common suggestion among all respondents (n = 4). Abbreviations: ^a^ = MS, medical student; ^b^ = FMG, foreign medical graduate.

## Data Availability

Survey respondents were assured raw data would remain confidential and would not be shared as when they consented to participate in this study.

## Appendix A Search Strategy Used to Identify Respondents

### Search Strategy Description:

A listing of 2020 US allopathic medical schools was obtained from the Association of American Medical Colleges (AAMC) website, members.aamc.org (https://members.aamc.org/eweb/DynamicPage.aspx?site=AAMC&webcode=AAMCOrgSearchResult&orgtype=Medical%20School (accessed on 1 October 2021)). A search was performed on April 5 2020 to identify student names associated with neurosurgical student organizations in all US allopathic medical schools by using the following search terms in Google: “Name of Medical School” + “Neurosurgery Student Group,” “Neurosurgery Interest Group,” “AANS.” Publicly available information on student names and emails was collected. Organization emails, Facebook pages, and websites for neurosurgical student organizations were also collected. The American Association of Neurological Surgeons (AANS) Medical Student Chapter directory and the 2020 Neurosurgical Residency Match list at aans.org (https://www.aans.org/Trainees/Medical-Students/Chapter-Directory, https://www.aans.org/Trainees/Medical-Students/Neurosurgical-Residency-Matches) (accessed on 1 October 2021) was consulted on April 5 2020 to further identify medical students interested in or transitioning to neurosurgery residency programs. A final internet search was performed and the emails of these students were collected. Three additional medical schools were identified via the AANS medical student chapters but were not on the AAMC website: University of Illinois College of Medicine Peoria, University of Illinois College of Medicine Rockford, and Mayo Clinic School of Medicine Arizona, these three schools were additionally searched.
